# Measurement of intraindividual airway tone heterogeneity and its importance in asthma

**DOI:** 10.1152/japplphysiol.00545.2015

**Published:** 2016-04-21

**Authors:** Robert H. Brown, Alkis Togias

**Affiliations:** ^1^Department of Anesthesiology and Critical Care Medicine, Johns Hopkins University, Baltimore, Maryland;; ^2^Department of Medicine, Division of Pulmonary and Critical Care Medicine, Johns Hopkins University, Baltimore, Maryland;; ^3^Department of Environmental Health Sciences, Division of Physiology, Johns Hopkins University, Baltimore, Maryland;; ^4^Department of Radiology, Johns Hopkins University, Baltimore, Maryland; and; ^5^Department of Medicine, Divisions of Allergy and Clinical Immunology and Pulmonary and Critical Care Medicine, Johns Hopkins University, Baltimore, Maryland

**Keywords:** airways, bronchodilation, distensibility

## Abstract

While airways have some degree of baseline tone, the level and variability of this tone is not known. It is also unclear whether there is a difference in airway tone or in the variability of airway tone between asthmatic and healthy individuals. This study examined airway tone and intraindividual airway tone heterogeneity (variance of airway tone) in vivo in 19 individuals with asthma compared with 9 healthy adults. All participants underwent spirometry, body plethysmography, and high-resolution computed tomography at baseline and after maximum bronchodilation with albuterol. Airway tone was defined as the percent difference in airway diameter after albuterol at total lung capacity compared with baseline. The amount of airway tone in each airway varied both within and between subjects. The average airway tone did not differ significantly between the two groups (*P* = 0.09), but the intraindividual airway tone heterogeneity did (*P* = 0.016). Intraindividual airway tone heterogeneity was strongly correlated with airway tone (*r* = 0.78, *P* < 0.0001). Also, it was negatively correlated with the magnitude of the distension of the airways from functional residual capacity to total lung capacity at both baseline (*r* = −0.49, *P* = 0.03) and after maximum bronchodilation (*r* = −0.51, *P* = 0.02) in the asthma, but not the healthy group. However, we did not find any relationship between intraindividual airway tone heterogeneity and conventional lung function outcomes. Intraindividual airway tone heterogeneity appears to be an important characteristic of airway pathophysiology in asthma.

airway smooth muscle tone is one of the most important characteristics that determine the state of the airway lumen, which, in turn, is considered the major factor behind symptoms of airway obstruction in asthma (5, 34). Bronchodilator-induced changes in airflow have been used as a surrogate in determining the magnitude of airway smooth muscle tone in vivo. However, because airflow is dependent on several other structural and functional characteristics of the airways and even lung parenchyma, we have used imaging through high-resolution computed tomography (HRCT) to determine bronchodilator-induced changes in the size of the airway lumen as a more direct surrogate of airway smooth muscle tone (9, 11). HRCT can determine the luminal diameters, as well as the airway wall thicknesses, in a large number of airways ranging from the trachea down to as small as 2 mm in diameter, and previous work by our group suggested that airway diameter and airway wall thickness may be important factors in airway tone (12). In addition, HRCT gives us the ability to measure the same airway locations on multiple occasions and under different physiological conditions. Using this approach, we have previously shown in both dogs (10) and in humans (12) that the smooth muscle of all airways has some degree of baseline tone and that the size of individual canine airways at baseline can vary widely over weeks or months (15) indicating the dynamic state of baseline tone.

In canine airways, when airway tone is increased with methacholine (Mch), the between-airway variability in airway size also increases (8) suggesting that tone is not distributed equally to all airways. This phenomenon is probably reflected in the ventilation heterogeneity that has been described in asthma using hyperpolarized noble gas MRI (17, 29, 37). In the case of HRCT, with individual airways being visualizable before and after the administration of a bronchodilator, between-airway heterogeneity of tone can be measured on the basis of the statistical variance around the mean change in airway luminal size.

We conducted this study to test the hypothesis that increased baseline tone, and increased heterogeneity in airway tone, both measured through airway imaging, are associated with asthma. We also hypothesized that increased airway wall thickness, a known characteristic of asthma, is associated with airway tone and airway tone heterogeneity. The specific aims of this study were *1*) to determine the magnitude of airway tone and the heterogeneity in airway tone in individuals with asthma compared with healthy controls; *2*) to examine whether intraindividual airway tone heterogeneity, compared with airway tone per se, best differentiates asthma from the healthy state; and *3*) to determine whether airway tone heterogeneity relates to lung function parameters or to airway diameter, wall thickness, and distensibility.

## METHODS

### 

#### Subjects.

The study involved a group of participants with asthma and a group of healthy controls. For the group with asthma, inclusion criteria were nonsmoker men or women between the ages of 18 and 65, who had typical asthma symptoms and history of exacerbations. To test a spectrum of disease, no asthma severity inclusion criteria were used, other than participant having no hospitalizations for asthma within the previous 6 mo. The presence of bronchial hyperresponsiveness to methacholine [concentration of methacholine leading to a fall in forced expired volume in 1 s (FEV_1_) of 20% (PC_20_) ≤ 8 mg/ml] and/or reversibility of airway obstruction by albuterol (an increase in FEV_1_ of >200 ml and ≥12% from the baseline or an increase ≥10% in predicted FEV_1_) were also used as asthma inclusion criteria. All asthma participants were at least 45 days past the end of their most recent upper respiratory tract infection for all of their visits and were asked to withhold inhaled bronchodilators prior to each study visit: 12 h for short-acting and 48 h for long-acting beta-agonists. The healthy group also included nonsmoker men and women between the ages of 18 and 65. Current or prior pulmonary symptoms or history of asthma or rhinitis were exclusionary for this group. The protocol was approved by the Johns Hopkins University Institutional Review Board, and written informed consents were obtained.

#### Study design.

The study consisted of three visits. The first visit was for screening purposes and included a respiratory questionnaire, epicutaneous skin testing with a panel of common aeroallergens, and baseline spirometry followed by a conventional methacholine (Mch) inhalation challenge (16a). During the second visit, subjects underwent spirometry and plethysmographic thoracic gas volume determination followed immediately by two sequential HRCT scans [one at functional residual capacity (FRC) and one at total lung capacity (TLC)] to evaluate baseline airway lumen size, wall thickness, and airway distensibility. During the third visit, after spirometry and lung volumes were again determined, all subjects received albuterol by nebulization until maximal bronchodilation was achieved (see description below). Thereafter, plethysmography was repeated followed by two HRCT scans at FRC and at TLC. For spirometry and body plethysmography outcomes, we used the Hankinson [Third National Health and Nutrition Examination Survey (NHANES III)] predicted values (22).

#### Evaluation of airway tone.

For the purpose of this study, we defined airway tone on the basis of imaging as the percent difference in airway diameter at baseline total lung capacity (TLC) compared with the diameter of the same airway after FEV_1_ plateau was reached with albuterol. Albuterol was delivered with at least two and up to four consecutive nebulizations of 2.5 mg in 3 ml of vehicle until FEV_1_ did not increase by more than 5% from its previous value (FEV_1_ plateau) (12).

Airway tone was defined by the following formula:

Airway tone=100−[(Airway diameter at TLC baseline)/(Airway diameter at TLC relaxed)]×100

An airway with no tone would be assigned the value of 0% with larger numbers indicating various levels of tone.

#### Evaluation of airway tone heterogeneity.

Intraindividual airway tone heterogeneity (variability) was defined as the statistical variance of airway tone for all the airways assessed within each subject.

#### Evaluation of airway distensibility at baseline and after maximum bronchodilation with albuterol.

As described above, subjects were scanned twice in sequence, once at FRC and once at TLC, both at baseline and after maximal bronchodilation with albuterol was achieved. Airway distensibility at baseline and after the FEV_1_ plateau was reached was defined by the following formula:

airway distensibility={[(Airway diameter at TLC)−(Airway diameter at FRC)]/(Airway diameter at FRC)}×100

where the airway diameter at TLC is an individual airway diameter measured on HRCT at full lung inflation and the airway diameter at FRC is in the same airway measured on HRCT at the end of normal expiration.

#### Acquisition of CT data.

All scans were performed using the spiral CT (Somatom Plus 4; Siemens) with settings of 120 kVp, 170 mA, 2-mm slice thickness, rotation feed of 2 mm/s, and a reconstruction interval of 1 mm (total 61 scans per set) during a single breath hold for ∼24 s at FRC or TLC and moved caudally. A reference scan was acquired prior to each spiral CT scan set to ensure reproducible image location in the lung. The images were reconstructed as 16-bit 512 × 512 matrix using a field of view of 200 mm. Images were reconstructed with the use of a high-spatial frequency (resolution) algorithm that enhanced edge detection, at a window level of −450 Hounsfield units (HU) and a window width of 1,350 HU. All airways visualized approximately perpendicular to the scan plane (long- to short-axis ratio less than 1.5:1) were assessed. For repeated airway measurements in a given study participant, adjacent anatomic landmarks, such as airway or vascular branching points, were identified on the HRCT images obtained at FRC and again on the TLC scans.

#### Airway measurements with HRCT.

The airway luminal size measurement has previously been described (6) and validated in animal models (2, 7). A similar range of airway generations (2nd through 6th) was evaluated in all the subjects. Briefly, the HRCT images were transferred to a UNIX-based workstation and analyzed using the airway analysis module of the Volumetric Image and Display Analysis (VIDA) software package (Department of Radiology, Division of Physiologic Imaging, University of Iowa, Iowa City, IA). To measure the airway areas, the operator drew a rough isocontour estimate of the lumen of the airway. The software program automatically located a precise isocontour perimeter of the airway lumen by sending out rays in a spoke wheel fashion to a predesignated pixel intensity level that defined the luminal edge of that airway wall. The length of the rays was set at 6 pixels. The software program used an algorithm for edge detection based on the “full-width-half-maximum” principle. The edge of the wall was defined by the program by the points along the lines where the pixel density changed to half its maximum through the wall. All full and partial pixels (full pixel size equals 0.1537 mm^2^ with our settings) within the adjusted isocontour were counted and represented the airway area.

To measure airway wall thickness, at least three lines were randomly drawn through the airway wall. The VIDA program displayed a histogram of pixel intensity along the line that was drawn through the airway wall. The inflection points of increasing intensity represented the inner and outer edges of the airway wall. The line and points were selected manually. The program then automatically measured the distance in pixels between the two points. The software is capable of measuring fractions of a pixel, and therefore the measurements of wall thickness were not necessarily quantized to multiples of the pixel dimension. The number of pixels and any fraction of a pixel that were counted in the airway wall were then converted to millimeters by multiplying by the size of the pixel in millimeters based on the scanning parameter. The airway wall measurements were then averaged to give a mean airway wall thickness for each airway. Airway wall thickness was expressed as a percentage of airway diameter measured at the FEV_1_ plateau. This was done to eliminate the potentially confounding effect of airway constriction on the apparent airway wall thickness.

#### Data analysis.

Data analysis was performed using JMP 11.0.0 software (SAS Institute). Unpaired *t*-tests were used to compare the two participant groups in terms of the two primary outcomes, airway tone and airway tone heterogeneity, as well as in terms of spirometric outcomes, lung volumes, airway diameter, and airway wall thickness measurements. To compare changes in pulmonary function after maximum bronchodilation with albuterol and changes in airway dimensions and airway wall thickness after maximum bronchodilation within and between the healthy and asthma groups, a mixed within-between groups ANOVA was used. Simple linear regressions were performed to measure the correlation between airway tone or airway tone heterogeneity and Mch reactivity (PC_20_), spirometric or lung volume outcomes, and outcomes of airway morphology. Logistic regressions were performed to examine the independent effects of airway tone and airway tone heterogeneity on the clinical phenotype (asthma and healthy status). Significance was accepted at *P* ≤ 0.05.

## RESULTS

We screened 44 subjects, 34 with histories consistent with asthma and 10 healthy controls. All 34 subjects with a history of asthma underwent albuterol reversibility testing, while 16 also underwent Mch bronchoprovocation. Methacholine challenge testing was not performed on seven asthma and two healthy subjects. In six asthma subjects, methacholine challenge testing was not performed because their baseline FEV_1_ was below 70% predicted (a safety requirement). One asthma and two healthy subjects refused methacholine challenge testing because of previous unpleasant experiences. Nineteen of the original 34 asthma participants fulfilled all inclusion criteria and were included in the main study (Table 1). Sixteen subjects were on inhaled beta-agonist bronchodilator, seven were on inhaled steroids, one was on nasal steroids, three were on oral steroids, three were on antihistamines, and two were on leukotriene receptor antagonists. One of the 10 healthy individuals developed a respiratory infection during the study, and his data were excluded from analysis (Table 1). Baseline and postalbuterol lung function data are shown in Table 2. Baseline and postalbuterol imaging-based airway measurements are shown in Table 3. Two of the healthy subjects demonstrated 12 and 13% airway reversibility, respectively, but this was not an exclusion criterion given that they had no history of asthma of rhinitis or any compatible symptoms.

**Table 1.** Baseline characteristics

Subject	Sex	Age	Race	FEV_1_, % pred	FVC, % pred	FEV_1_/FVC Baseline	PC_20_, mg/ml	Reversibility (% Δ From Baseline)	RV, % pred	TLC, % pred	RV/TLC
*Asthma subjects*											
*A1*	M	25	AA	75	97	66	0.22	30	179	131	0.40
*A2*	M	23	AA	104	121	74	23.39	12	104	108	0.25
*A3*	M	35	AA	68	111	51	#	54	#	#	#
*A4*	M	23	C	90	104	71	1.5	18	220	164	0.32
*A5*	M	33	C	72	87	67	1.61	24	98	128	0.28
*A6*	F	28	AA	88	110	69	0.02	12	63	144	0.18
*A7*	F	25	C	85	98	75	2.63	8	135	124	0.44
*A8*	M	38	C	85	97	70	#	16	283	193	0.43
*A9*	F	44	AA	75	89	69	2.14	16	#	#	#
*A10*	F	22	C	109	121	79	>25	16	#	#	#
*A11*	M	28	HSP	83	94	75	5.76	3	98	95	0.24
*A12*	M	43	AA	75	80	76	4.109	6	100	73	0.38
*A13*	F	48	AA	69	73	77	#	21	124	85	0.52
*A14*	F	34	AA	87	87	84	0.58	8	303	140	0.64
*A15*	M	60	C	60	82	56	#	12	173	110	0.51
*A16*	M	34	AA	43	73	49	#	22	#	#	#
*A17*	F	50	AA	69	75	74	#	15	111	53	0.60
*A18*	M	45	AA	43	94	37	#	22	234	150	0.50
*A19*	F	19	C	74	83	79	0.45	7	93	87	0.23
Mean ± SD		35 ± 11		77 ± 17	93 ± 15	68 ± 12		17 ± 11	154 ± 74	119 ± 37	0.039 ± 0.14
*Healthy subjects*											
*H1*	M	21	C	81	100	68	>25	12	97	116	0.17
*H2*	M	52	C	86	97	69	>25	4	100	101	0.30
*H3*	F	20	C	89	84	93	>25	4	119	89	0.32
*H4*	F	23	C	82	86	82	>25	13	#	#	#
*H5*	M	47	C	81	89	71	>25	1	147	97	0.44
*H6*	M	41	C	111	116	75	#	5	88	111	0.22
*H7*	M	20	HSP	88	93	81	>25	5	94	107	0.18
*H8*	M	27	C	102	102	81	#	0	85	107	0.19
*H9*	F	35	C	101	110	78	12.9	8	88	88	0.30
Mean ± SD		32 ± 12		91 ± 11	98 ± 11	78 ± 8		4 ± 4	107 ± 25	101 ± 10	0.28 ± 0.1

FVC, forced vital capacity; RV, residual volume; % pred, ratio of actual results compared to predicted normal values, expressed as a percentage; M, male; F, female; AA, African American; C, Caucasian; HSP, Hispanic.

# Not performed.

**Table 2.** Pulmonary function

Measurement	Healthy Controls (*n* = 9)	Asthma (*n* = 19)	Between Groups *t*-Test *P* Value
*Baseline*			
FEV_1_, % pred	91 ± 10.6%	77 ± 17%	0.03*
FVC, % pred	98 ± 10.7%	93 ± 15%	0.47
FEV_1_/FVC	0.78 ± 0.08	0.68 ± 0.12	0.05*
RV, % pred	102 ± 21%	155 ± 74%	0.07
FRC, % pred	124 ± 27%	129 ± 38%	0.77
TLC, % pred	102 ± 10%	108 ± 32%	0.60
RV/TLC	0.27 ± 0.09%	0.40 ± 0.12%	0.03*
*Change after maximal relaxation with albuterol*			
% ΔFEV_1_	5 ± 5% (*P* = 0.17)	17 ± 11% (*P* < 0.0001)*	0.02*
% ΔFVC	−0.5 ± 2.2% (*P* = 0.97)	6 ± 6% (*P* < 0.0002)*	0.09
% ΔFEV_1_/FVC	6 ± 4% (*P* = 0.03)*	10 ± 8% (*P* < 0.0001)*	0.07
% ΔRV	4 ± 18% (*P* = 0.99)	−15 ± 24% (*P* = 0.01)*	0.21
% ΔFRC	−8 ± 8% (*P* = 0.18)	−9 ± 10% (*P* = 0.01)*	0.69
% ΔTLC	2 ± 3% (*P* = 0.73)	−5 ± 6% (*P* = 0.009)*	0.36
% ΔRV/TLC	2 ± 15% (*P* = 0.99)	−11 ± 20% (*P* = 0.07)	0.06

Values are means ± SD. *P* values within groups represent paired comparisons of baseline vs. postalbuterol maximum relaxation measurements using a mixed within-between groups ANOVA.

* *P* < 0.05.

**Table 3.** Airway dimensions

Measurement	Healthy Controls (*n* = 9)	Asthma (*n* = 19)	Between Groups *t*-Test (*P* Value)
*Baseline*			
Baseline diameter FRC	5.19 ± 0.64	5.06 ± 1.0	0.74
Baseline diameter TLC	5.91 ± 0.83	5.68 ± 1.3	0.63
Wall thickness (fraction of diameter) FRC	0.24 ± 0.04	0.31 ± 0.06	0.006*
Wall thickness (fraction of diameter) TLC	0.19 ± 0.03	0.25 ± 0.07	0.03*
% Δ diameter from FRC to TLC baseline	16 ± 8%	14 ± 11%	0.62
*After maximum relaxation*			
Relaxed diameter FRC	6.4 ± 0.8	6.2 ± 1.0	0.53
Relaxed diameter TLC	6.6 ± 0.9	6.6 ± 1.1	0.91
% Δ diameter at FRC (baseline to relaxed)	27 ± 7% (*P* < 0.0001)*	27 ± 16% (*P* < 0.0001)*	0.62†
% Δ diameter at TLC (baseline to relaxed) (defined as airway tone)	13 ± 3% (*P* < 0.0001)*	22 ± 17% (*P* < 0.0001)*	0.83†
Wall thickness (fraction of diameter) FRC after relaxation	0.18 ± 0.03	0.22 ± 0.03	0.002*
Wall thickness (fraction of diameter) TLC after relaxation	0.16 ± 0.02	0.19 ± 0.03	0.03*
% Δ wall thickness (fraction of diameter) FRC (baseline to relaxed)	24.4 ± 4.8 (*P* < 0.0001)*	24.9 ± 14 (*P* < 0.0001)*	0.11
% Δ wall thickness (fraction of diameter) TLC (baseline to relaxed)	14.4 ± 3.6 (*P* < 0.0001)*	21.8 ± 13 (*P* < 0.0001)*	0.92
% Δ diameter from FRC to TLC relaxed	3 ± 4%	10 ± 9%	0.046*

Values are means ± SD. *P* values within groups represent paired comparisons of baseline vs. postalbuterol maximum relaxation measurements.

* *P* < 0.05.

† Analysis by mixed within-between groups ANOVA.

### 

#### Tone.

**Fig. 1.**
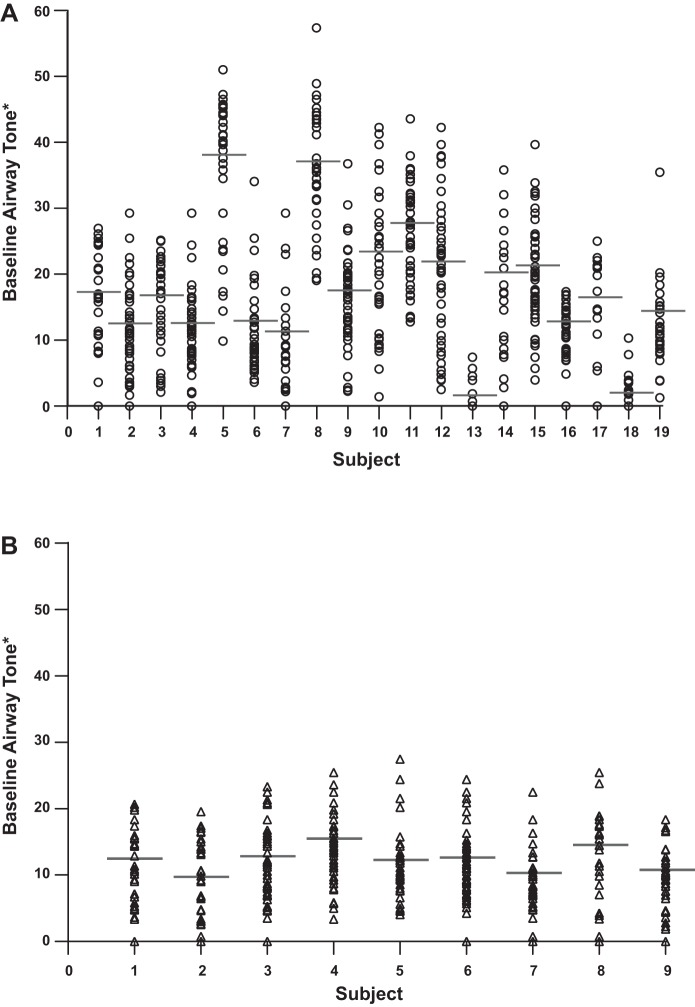
Baseline airway tone for all measured airways in each individual with asthma (*A*) and healthy controls (*B*). Each column represents one subject, and each circle or triangle represents one airway. The thick horizontal line represents mean airway tone for that subject. An airway in which albuterol did not change airway diameter is depicted with “zero” tone. *Baseline airway tone is defined as 100 − [(Airway diameter at TLC baseline)/(Airway diameter at TLC relaxed)] × 100.

**Fig. 2.**
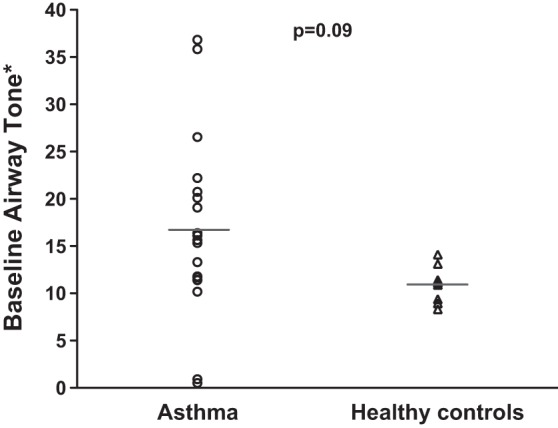
Baseline airway tone in the asthma and the healthy groups. ○, mean airway tone for all airways in each study participant in the asthma group; △, mean airway tone for all airways in each healthy participant. The horizontal lines represent the mean airway tone for all the subjects in each group. No significant difference between the two groups was found (*P* = 0.09). *Baseline airway tone is defined as 100 − [(Airway diameter at TLC baseline)/(Airway diameter at TLC relaxed)] × 100.

Airway tone, as defined in methods, was calculated for each volunteer by averaging the tone of all measurable airways (mean number of airways measured equals 40, range 18-64) (Fig. 1, *A* and *B*). A trend for higher airway tone was seen in asthma participants, but the difference between the two study groups did not reach significance (*P* = 0.088, Fig. 2).

#### Intraindividual tone heterogeneity.

**Fig. 3.**
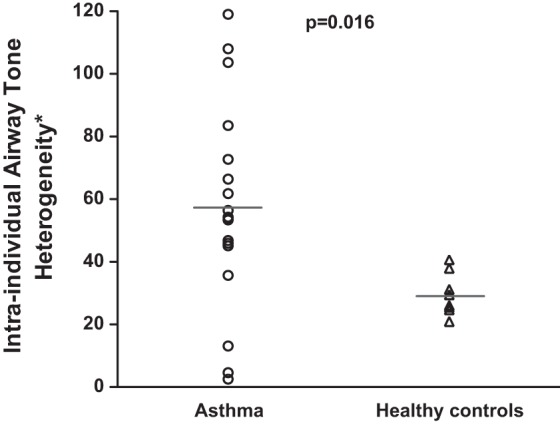
Intraindividual airway tone heterogeneity in the asthma and the healthy groups. ○, mean intraindividual airway tone heterogeneity for all airways in each study participant in the asthma group; △, mean airway tone for all airways in each healthy participant. The horizontal lines represent the mean intraindividual airway tone heterogeneity for all the subjects in each group. A significant difference in intraindividual airway tone heterogeneity between the two groups was found (*P* = 0.016). *Intraindividual airway tone heterogeneity is defined as the statistical variance of airway tone for all the airways assessed within each subject.

Intraindividual airway tone heterogeneity (variability) was calculated as the variance around the mean airway tone for all the airways within each subject (see methods). We found that the intraindividual tone heterogeneity was significantly higher in asthma participants compared with healthy controls (*P* = 0.016; Fig. 3). We also performed logistic regression with “health status” (asthma or health) as the outcome variable and with intraindividual airway tone heterogeneity as the independent variable. Again, intraindividual airway tone heterogeneity was predictive of asthma (*P* = 0.0067).

Intraindividual airway tone heterogeneity was strongly correlated with airway tone (*r* = 0.78, *P* < 0.0001). Therefore we performed logistic regression with “health status” (asthma or health) as the outcome variable and airway tone and intraindividual airway tone heterogeneity as the independent variables. In addition, we included an interaction term for these two variables in the model. The overall model was statistically significant (*P* = 0.0003). However, only intraindividual airway tone heterogeneity showed an association (*P* = 0.03) while airway tone did not (*P* = 0.27). The interaction term added ∼10% to the main effect of the intraindividual tone heterogeneity.

#### Relation of intraindividual airway tone heterogeneity or airway tone with lung function and CT-based airway measurements.

**Fig. 4.**
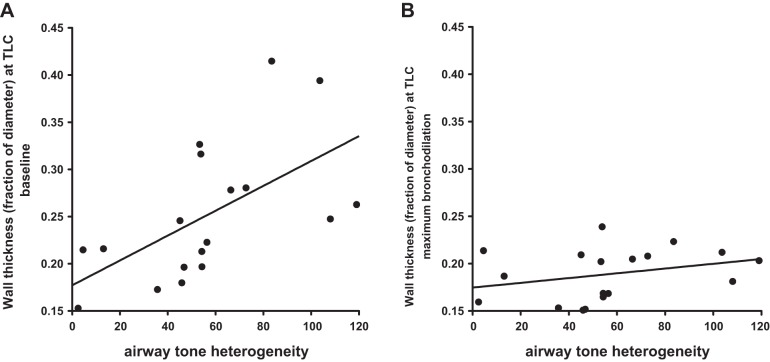
Relationship between airway wall thickness (as a fraction of airway diameter) at TLC and airway tone heterogeneity. *A*: baseline. *B*: after maximum bronchodilation with albuterol. These figures clearly demonstrate that the statistically significant correlation between wall thickness and heterogeneity at baseline (*P* = 0.01) is eliminated after maximum bronchodilation (*P* = 0.22). These results strongly support the notion that the relationship at baseline is due to the presence of airway tone.

We examined the relationships between intraindividual airway tone heterogeneity and pulmonary function (Table 4) or imaging-based airway measurements (Table 5) at baseline and after albuterol. In the group of participants with asthma, intraindividual airway tone heterogeneity correlated with baseline airway diameter at TLC (*r* = −0.54, *P* = 0.02) and with airway wall thickness at both FRC (*r* = 0.48, *P* = 0.04) and TLC (*r* = 0.59, *P* = 0.01) (Table 5 and Fig. 4*A*). These relationships indicated that among our study participants with asthma, those with narrower airways and thicker airway walls had greater airway tone heterogeneity. After albuterol, these relationships were lost. Figure 4*B* shows that the relationship between airway tone heterogeneity and airway wall thickness was lost because individuals whose airways had the greatest average wall thickness at baseline also had the greatest reductions in wall thickness with maximum bronchodilation, whereas airways with the lowest wall thickness had relatively little change. Interestingly, the change in wall thickness with maximum bronchodilation was strongly correlated with airway tone (*r* = 0.97, *P* < 0.0001). Intraindividual airway tone heterogeneity did not correlate with any baseline measures of spirometry or body plethysmography in either the asthma or healthy control groups nor did it correlate with the albuterol-induced changes in those parameters (Table 4).

**Table 4.** Correlations between intraindividual airway tone heterogeneity and pulmonary function

	Asthma	Health
Baseline FEV_1_, % pred	*r* = 0.18, *P* = 0.45	*r* = 0.11, *P* = 0.77
Baseline FVC, % pred	*r* = 0.17, *P* = 0.48	*r* = 0.12, *P* = 0.76
Baseline FEV_1_/FVC	*r* = −0.15, *P* = 0.54	*r* = −0.15, *P* = 0.69
Baseline RV, % pred	*r* = −0.16, *P* = 0.58	*r* = −0.23, *P* = 0.58
Baseline TLC, % pred	*r* = −0.13, *P* = 0.65	*r* = 0.13, *P* = 0.77
Baseline RV/TLC	*r* = −0.17, *P* = 0.53	*r* = −0.14, *P* = 0.73
Postalbuterol FEV_1_, % pred	*r* = 0.29, *P* = 0.23	*r* = 0.00, *P* = 0.99
Postalbuterol FVC, % pred	*r* = 0.24, *P* = 0.33	*r* = 0.08, *P* = 0.84
Postalbuterol FEV_1_/FVC	*r* = 0.24, *P* = 0.32	*r* = −0.26, *P* = 0.49
Postalbuterol RV, % pred	*r* = −0.11, *P* = 0.69	*r* = −0.44, *P* = 0.28
Postalbuterol TLC, % pred	*r* = −0.15, *P* = 0.60	*r* = 0.07, *P* = 0.87
Postalbuterol RV/TLC	*r* = −0.05, *P* = 0.87	*r* = −0.15, *P* = 0.72

**Table 5.** Correlations between intraindividual airway tone heterogeneity and airway measurements

	Asthma	Health
Diameter baseline FRC	*r* = −0.42, *P* = 0.07	*r* = 0.48, *P* = 0.20
Diameter baseline TLC	*r* = −0.54, *P* = 0.02*	*r* = 0.30, *P* = 0.44
Diameter postalbuterol FRC	*r* = −0.04, *P* = 0.87	*r* = 0.21, *P* = 0.60
Diameter postalbuterol TLC	*r* = −0.24, *P* = 0.33	*r* = 0.35, *P* = 0.35
WT (frac dia) baseline FRC	*r* = 0.48, *P* = 0.04*	*r* = −0.31, *P* = 0.42
WT (frac dia) baseline TLC	*r* = 0.59, *P* = 0.01*	*r* = −0.34, *P* = 0.36
WT (frac dia) postalbuterol FRC	*r* = −0.13, *P* = 0.61	*r* = −0.25, *P* = 0.52
WT (frac dia) postalbuterol TLC	*r* = 0.30, *P* = 0.22	*r* = −0.43, *P* = 0.25
% Δ diameter FRC-TLC baseline	*r* = −0.49, *P* = 0.03*	*r* = −0.14, *P* = 0.72
% Δ diameter FRC-TLC postalbuterol	*r* = −0.51, *P* = 0.02*	*r* = 0.50, *P* = 0.17
% Δ diameter TLC baseline to postalbuterol (tone)	*r* = 0.78, *P* < 0.001*	*r* = 0.09, *P* = 0.82

WT (frac dia), airway wall thickness expressed as a fraction of airway diameter.

* *P* < 0.05.

We also examined the relationships between airway tone and pulmonary function (Table 6) and airway measurements (Table 7). There were no significant correlations between airway tone and pulmonary function at baseline or between airway tone and the albuterol-induced changes in spirometry or plethysmography. However, we found significant correlations between airway tone and airway diameter at baseline FRC (*r* = −0.59, *P* = 0.008), airway diameter at baseline TLC (*r* = −0.72, *P* = 00005), wall thickness at baseline FRC (*r* = 0.70, *P* = 0.0012), and wall thickness at baseline TLC (*r* = 0.85, *P* < 0.0001) in participants with asthma. As with airway tone heterogeneity, all these relationships were lost after maximum bronchodilation (Table 7). In participants with asthma, airway tone correlated with Mch reactivity as assessed by the log PC_20_ (*r* = −0.61, *P* = 0.046), where greater tone was associated with lower PC_20_. Interestingly, there was no correlation between the PC_20_ and airway tone heterogeneity (*r* = −0.04, *P* = 0.91).

**Table 6.** Correlations between airway tone and pulmonary function

	Asthma	Health
Baseline FEV_1_, % pred	*P* = 0.56	*P* = 0.99
Baseline FVC, % pred	*P* = 0.43	*P* = 0.57
Baseline FEV_1_/FVC	*P* = 0.75	*P* = 0.32
Baseline RV, % pred	*P* = 0.32	*P* = 0.93
Baseline TLC, % pred	*P* = 0.24	*P* = 0.63
Baseline RV/TLC	*P* = 0.59	*P* = 0.72
Postalbuterol FEV_1_, % pred	*P* = 0.22	*P* = 0.78
Postalbuterol FVC, % pred	*P* = 0.23	*P* = 0.59
Postalbuterol FEV_1_/FVC	*P* = 0.43	*P* = 0.15
Postalbuterol RV, % pred	*P* = 0.51	*P* = 0.10
Postalbuterol TLC, % pred	*P* = 0.16	*P* = 0.92
Postalbuterol RV/TLC	*P* = 0.71	*P* = 0.19

**Table 7.** Correlations between airway tone and airway measurements

	Asthma	Health
Diameter baseline FRC	*r* = −0.59, *P* = 0.008*	*P* = 0.39
Diameter baseline TLC	*r* = −0.72, *P* = 0.0005*	*P* = 0.13
Diameter postalbuterol FRC	*P* = 0.53	*P* = 0.16
Diameter postalbuterol TLC	*P* = 0.15	*P* = 0.27
WT (frac dia) baseline FRC	*r* = 0.70, *P* = 0.0012*	*P* = 0.82
WT (frac dia) baseline TLC	*r* = 0.85, *P* < 0.0001*	*P* = 0.78
WT (frac dia) postalbuterol FRC	*P* = 0.72	*P* = 0.81
WT (frac dia) postalbuterol TLC	*P* = 0.11	*P* = 0.75
% Δ diameter FRC-TLC baseline	*r* = −0.60, *P* = 0.007*	*r* = −0.71, *P* = 0.03*
% Δ diameter FRC-TLC postalbuterol	*r* = −0.49, *P* = 0.03*	*r* = 0.21, *P* = 0.59

WT (frac dia), airway wall thickness expressed as a fraction of airway diameter.

* *P* < 0.05.

Deep inspirations are thought to be “defective” in protecting or reversing airway constriction in persons with asthma. In the asthma group, we found negative correlations between intraindividual airway tone heterogeneity and the distension of the airways from FRC to TLC with a deep inspiration at both baseline (*r* = −0.49, *P* = 0.03) and after maximum bronchodilation (*r* = −0.51, *P* = 0.02) (Table 5) with greater intraindividual tone heterogeneity associated with less airway distention from FRC to TLC. We found no correlation between intraindividual airway tone heterogeneity and distension of the airways with deep inspiration in the healthy group.

Finally, we examined the relationships between airway tone and distensibility. As with intraindividual airway tone heterogeneity, we found negative correlations between airway tone and the distension of the airways from FRC to TLC with a deep inspiration at both baseline (*r* = −0.60, *P* = 0.007) and after maximum bronchodilation with albuterol (*r* = −0.49, *P* = 0.03) in the asthma group (Table 7). We also found a correlation between airway tone and distension of the airways with deep inspiration at baseline in the healthy group (*r* = −0.71, *P* = 0.03).

## DISCUSSION

In this study, we used airway imaging to quantify airway tone and intraindividual airway tone heterogeneity in participants with asthma of variable severity and in healthy individuals. We found that, compared with the healthy group, individuals with asthma have larger baseline intraindividual airway tone heterogeneity. On the other hand, averaging the tone of multiple airways within each participant did not yield a statistically significant difference between the asthma and the healthy groups, but a clear trend was present for asthma to have higher tone, and the lack of statistical significance may reflect sample size. We found that airway tone and airway tone heterogeneity correlated with airway luminal diameter and airway wall thickness at baseline, but not after maximum bronchodilation. On the other hand, both airway tone and heterogeneity correlated with airway distensibility from FRC to TLC both at baseline and after maximum bronchodilation with albuterol. We did not find any relationships between airway tone or airway tone heterogeneity and measures of pulmonary function assessed by spirometry or body plethysmography.

Measuring airway smooth muscle tone in vivo is a difficult task. One intuitively assumes that changes in airflow parameters induced by bronchodilators or by a spasmogenic agent like inhaled methacholine reflect changes in airway tone. This is true only to some extent because airflow is dependent on several factors including airway resistance, tissue resistance, elastic recoil, and lung volume. Furthermore, using spirometry to assess airflow characteristics before and after the administration of a bronchodilator or before and after the administration of a spasmogen introduces the effects of deep inspirations on the state of airway smooth muscle, which can influence tone (18). Measuring airway diameter with the use of HRCT before and after maximal bronchodilation with a beta-adrenergic agonist offers a different, more direct approach to quantifying tone. We have previously applied this methodology in a canine model (9), where we showed that after systemic administration of atropine at a dose that completely blocks vagal tone, airway diameters reach a plateau at very low transpulmonary pressure and further increases in pressure lead to negligible changes in diameter. With administration of aerosolized histamine, airway diameters were reduced and did not reach maximum relaxed size with increased transpulmonary pressure. In the current study, given the limitations of working with human volunteers, removal of airway tone was achieved with repeated doses of inhaled albuterol until a plateau in FEV_1_ was reached. In addition, to ensure maximal physiologic transpulmonary pressure, we used the airway diameter at TLC as the primary outcome to assess changes from baseline. Limitations of this model include the possibility that smaller airways may not have been reached by inhaled albuterol and that smaller airways cannot be adequately visualized by HRCT. We can, however, argue that our measurements were obtained on conducting airways that were most likely reachable by albuterol and maximally relaxed.

Our data indicate that airway tone intraindividual heterogeneity was a strong characteristic of asthma, perhaps stronger than airway tone per se. As we have previously seen in the canine model, where induction of airway tone led to increased airway heterogeneity (8), we found that airway tone intraindividual heterogeneity was strongly correlated to airway tone, but when both parameters were tested in a single predictive model, only intraindividual airway tone heterogeneity was associated with the diagnosis of asthma. We note, however, that within the asthma group, airway tone did correlate with bronchial responsiveness to methacholine (PC_20_), whereas intraindividual tone heterogeneity did not, indicating that each parameter may play a different role in the behavior of the airways in the context of asthma. It should be noted that not all subjects underwent the methacholine PC_20_ provocation test because of various reasons. Whether the inclusion of all the subjects would have changed the results is unknown.

All individuals with asthma enrolled in this study had a clinical history of asthma, used asthma medications, and showed airway hyperresponsiveness by either bronchoconstriction to methacholine and/or bronchodilation to albuterol. All healthy subjects had no current or prior pulmonary symptoms or history of asthma or rhinitis. In retrospect, three subjects were identified (*asthma subjects A8* and *A17* and *healthy subject H1*, Table 1) whose lung function testing could raise questions as to the correct classification. After excluding these three subjects, there still was no significant difference in airway tone between the asthma and healthy groups (*P* = 0.15), whereas airway tone heterogeneity remained significantly different (*P* = 0.038).

Over the years, the potential role of airway heterogeneity in health and disease has attracted substantial interest (3, 19, 20, 23–25, 27, 28, 31, 32, 38–44). For example, King et al. used HRCT to examine the variability of the airway response to inhaled methacholine in subjects with asthma (28). Indeed, most studies have examined the heterogeneity of airway responsiveness to a spasmogen, and several have used HRCT or PET imaging to assess airway narrowing. We are not aware of another study examining the heterogeneity of the bronchodilator response using imaging. Another method of estimating global heterogeneity in the lung is with lung impedance (19, 20, 27, 32). The degree of heterogeneity is based on modeling the changes in the frequency dependence of lung resistance and elastance. With this approach it has been shown that heterogeneity is higher in people with more severe asthma, compared with moderate asthma and healthy controls (26, 32). However, this method cannot provide insight into the spatial or anatomic details underlying heterogeneity. These models, when combined with imaging techniques, can improve estimates regarding which diameters are involved in producing heterogeneity of ventilation (39). A few studies have been performed in children using impedance measurements, and their findings support our results. For example, in clinically stable children with asthma, Goldman et al. (21) demonstrated day-to-day variability in respiratory impedance not detected by spirometry.

Relevant to our findings and a key aspect of asthma is the patchiness of lung ventilation during constriction as well as during quiescent times when symptoms are absent (37). Airway tone heterogeneity may be a major factor in the patchiness that defines asthma. Leary et al. and Venegas and colleagues showed that even for uniform smooth muscle activation of a symmetric bronchial tree, the presence of minimal heterogeneity breaks the symmetry and leads to large clusters of poorly ventilated lung units (30, 38, 42), the hallmark of asthma. Our findings are consistent with this potential mechanism of asthma symptoms. The mechanism behind increased heterogeneity in the behavior of the airways remains to be determined. If one considers all the factors that play into airway tone—central or peripheral drive, activation of preganglionic nerves, synaptic transmission in the parasympathetic ganglia, prejunctional regulation of acetylcholine release, muscle contraction—and if one also realizes that each airway is likely driven by distinct subsets of these nerves, all of this sets up the possibility that heterogeneity of airway caliber and responses could be easily influenced by continual fluctuations in parasympathetic/cholinergic drive (16).

One of the aims of this study was to examine the associations between airway tone or intraindividual airway tone heterogeneity and baseline spirometry or plethysmography and the changes induced by albuterol on those outcomes. We were not surprised to find that our measure of baseline airway tone did not correlate with baseline spirometric or plethysmographic outcomes or with the albuterol-induced changes on those outcomes. As discussed earlier, spirometric outcomes are influenced by several mechanical and volume factors that seem to be playing an important role at baseline, in addition to airway tone. Furthermore, small airways contribute to airway resistance and to air trapping and increased residual volume, but HRCT imaging cannot assess the geometry of those airways. In some of our previous studies (4, 13, 36), pulmonary function test values did correlate with CT measurements, but those studies were usually performed in settings where there were large changes in pulmonary function test values, most of the time after induced airway constriction. We have also reported lack of a relationship between physiologic measurements and imaging outcomes in dogs (14). In those studies, lack of correlation between airway size and lung function measurements after pharmacologically induced airway relaxation was due to the fact that the changes in diameter with relaxation were relatively small compared with the changes in lung volume that were associated with airflow changes.

Another aim was to assess whether geometrical aspects of the airways were associated with airway tone or with intraindividual airway tone heterogeneity. Airway wall thickness expressed as a fraction of airway diameter correlated with airway tone as well as with intraindividual airway tone heterogeneity at baseline (Fig. 4*A*), but not after albuterol (Fig. 4*B*). This suggests that the statistically significant relationships were secondary to the presence of airway tone at baseline. Notably, albuterol significantly reduced airway wall thickness indicating that tone contributes to wall thickness (Table 3), probably through the principle of mass conservation with airway narrowing. Furthermore, the loss of the relationship between airway wall thickness and airway tone heterogeneity after albuterol (Fig. 4*B*) was due to the fact that the airways with the thickest walls at baseline showed the largest reductions in wall thickness after maximal bronchodilation. We found a significant difference between the asthma and the healthy groups, in wall thickness, even after removal of tone with albuterol (Table 3) indicating that remodeling was, indeed, present in our asthma group. However, our data suggest that remodeling is not a major contributor to airway tone or airway tone heterogeneity.

Airway distensibility, the change in airway diameter from FRC to TLC, which is one of the determinants of deep inspiration-induced bronchodilation and is reduced in magnitude in severe asthma (35), was negatively correlated with intraindividual airway tone heterogeneity and airway tone in asthma. This supports the concept that increased tone stiffens the airways and reduces their ability to distend. However, these relationships remained present even when distensibility was measured after maximum bronchodilation with albuterol. This raises the possibility that airway remodeling also contributes to reduced airway distensibility in some people with asthma.

In summary, we have identified intraindividual airway tone heterogeneity, measured by the variance of the change of airway diameters in response to albuterol, as a parameter strongly associated with the diagnosis of asthma independent of conventional lung function measurements. Our finding is consistent with the previous observations of lung ventilation inhomogeneity in asthma. We believe that this observation and the methodology we have applied can be used to further explore airway pathophysiology in asthma.

## GRANTS

This work was supported by National Institutes of Health Grants RO1-HL-61277, R01-HL-62698, and RO1-HL-10342.

## DISCLOSURES

No conflicts of interest, financial or otherwise, are declared by the author(s).

## AUTHOR CONTRIBUTIONS

R.H.B. conception and design of research; R.H.B. performed experiments; R.H.B. and A.T. analyzed data; R.H.B. and A.T. interpreted results of experiments; R.H.B. prepared figures; R.H.B. and A.T. drafted manuscript; R.H.B. and A.T. edited and revised manuscript; R.H.B. and A.T. approved final version of manuscript.
